# Pain intensity, neck pain and longer duration of complaints predict poorer outcome in patients with shoulder pain – a systematic review

**DOI:** 10.1186/s12891-015-0738-4

**Published:** 2015-10-09

**Authors:** Margit K. Kooijman, Di-Janne A. Barten, Ilse CS Swinkels, Ton Kuijpers, Dinny de Bakker, Bart W. Koes, Cindy Veenhof

**Affiliations:** NIVEL Netherlands Institute of Health Services Research, Utrecht, The Netherlands; Dutch College of General Practitioners, Utrecht, The Netherlands; TRANZO Tilburg University, Tilburg, The Netherlands; Erasmus Medical Center, Rotterdam, The Netherlands; University Medical Center, Utrecht, The Netherlands

**Keywords:** Shoulder, Pain, Musculoskeletal disease, Chronic pain, Prognosis, Review

## Abstract

**Background:**

Shoulder complaints are common and have an unfavourable prognosis in many patients. Prognostic information is helpful for both patients and clinicians in managing the complaints. The research question was which factors have prognostic value on (un)favourable outcome in patients with shoulder complaints in primary care, secondary care and occupational settings.

**Methods:**

Update of a systematic review in primary care, secondary care and occupational settings.

**Results:**

Nine articles were published since the original review in 2004. Six were of high quality covering a wide variety of prognostic factors and outcome measures. Four studies were conducted in primary care settings. A best evidence synthesis, including the results of the previous systematic review on this topic shows that there is strong evidence that higher shoulder pain intensity, concomitant neck pain and a longer duration of symptoms predict poorer outcome in primary care settings. In secondary care populations, strong evidence was found for the association between greater disability and poorer outcome and between the existence of previous shoulder pain and poorer outcome.

**Conclusion:**

Clinicians may take these factors into account in the management of their patients. Those with a worse prognosis may be monitored more frequently and the treatment plan modified if complaints persist.

**Electronic supplementary material:**

The online version of this article (doi:10.1186/s12891-015-0738-4) contains supplementary material, which is available to authorized users.

## Background

Shoulder complaints are common in the general population. A systematic review by Luime et al. (2004) indicates that prevalence figures range from 7 to 26 % for point prevalence, up to 67 % for lifetime prevalence [1]. In the Netherlands, the annual consulting incidence in general practice for shoulder symptoms is estimated at 29 per 1000 person years [2]. In physiotherapy practice, 9.8 % of patients present themselves with shoulder complaints which makes it the most common complaint of the extremities [3].

 From previous studies, it is known that there is an unfavourable long-term outcome in many patients with shoulder complaints [4, 5]. This is troublesome for patients as well as clinicians and in time for employers and insurance. Although treatment of patients with shoulder problems is mainly an issue for primary care [6], previous research shows that a relatively small group of patients is responsible for high costs for secondary care and sick leave, which accounts for a large part of total costs of shoulder pain [7]. To optimize the treatment of shoulders complaints, it is helpful to obtain insight into prognostic factors related to shoulder complaints. Prognostic information is important for clinicians to identify patients with a higher risk for developing chronic pain or disability. When shown robust and modifiable, this information can facilitate clinical decision-making and if necessary, timely and specific consultation with or referral to other health care providers. For patients, it can provide adequate knowledge about the expected course of their shoulder problems and facilitate adequate coping with them.

In 2004, a systematic review was published on prognostic studies on shoulder disorders [8]. It included six high quality and ten low quality studies, mostly performed in a secondary care setting. The review reported strong evidence that high pain intensity predicts a poorer outcome in primary care populations and that middle age predicts poorer outcome in occupational populations. Moderate evidence was found for long duration of complaints and high disability at baseline as predictors of poorer outcome in primary care. Because the results were based on a small number of studies and the majority was conducted in secondary care, they need to be interpreted with caution. Because new studies, especially in the primary care setting, have been published on predictors of outcome we decided to update the evidence on prognostic factors on the outcome of shoulder disorders. The research question was which factors have prognostic value on (un)favourable outcome in patients with shoulder complaints in primary care, secondary care and occupational setting.

## Methods

### Search strategy

This review updates previous work by Kuijpers et al. (2004) [8]. Therefore, a computerized literature search was performed in PubMed and Embase using the same search strategy with the exception that the search was confined to the dates February 2003 through February 2014. Some key words and/or medical subject headings changed hence the following search terms were used: shoulder/abnormalities, shoulder/injuries, shoulder/pathology, shoulder/physiopathology. shoulder pain, shoulder joint, shoulder impingement syndrome, clinical study, longitudinal study, intervention study, cohort studies, prospective study, retrospective study, incidence, mortality, prognos*, predict*, course. Selection criteria were adopted from Kuijpers et al. (2004) [8]:

- The study focussed on patients suffering from shoulder complaints
- The association of at least one prognostic factor with the outcome of shoulder pain had to be presented
- The design had to be a cohort study
- The article was published in English
- Results were published as a full report before February 2014
- Studies that focused on shoulder pain due to luxation, cancer or systematic diseases such as rheumatoid arthritis or osteoporosis were excluded. Also studies that focused on the results of surgery were excluded.

Additionally, a manual search was conducted to retrieve relevant publications from the reference lists of all selected publications. Two authors (MK and DB) read titles, abstracts and full-text articles. Studies were excluded if the content did not meet the inclusion criteria. Disagreements regarding article inclusion were resolved by discussion between the two reviewers. If consensus could not be reached, a third reviewer (IS) was consulted and had the final vote.

### Quality assessment

Three reviewers (MK, IS, CV) independently assessed the methodological quality of each article using the checklist designed and used by Kuijpers et al. (2004) (Table 1) [8]. The checklist covers aspects of internal validity (criteria A, D, E, F, G, H, I, J, K, L, M, P, Q), generalisability (criteria B, C, N, O) and precision (criterion R) (Additional file 1). It contains seven categories: study population, response rate, follow-up, treatment, outcome, prognostic factors and data presentation. The list contains 18 criteria that can be scored positive (‘+’), negative (‘-‘) or unclear (‘?‘). The total score is the sum of all the criteria that are scored positive. The cut-off point used by Kuijpers et al. (2004) which was shown to be robust, was adopted; studies with scores > 8 points (>60 % of the maximum attainable score) were considered to be of high quality, studies that scored ≤ 8 points of low quality [8]. Disagreements between reviewers on study quality were resolved by discussion between the three reviewers.

**Table 1:** Criteria list for assessing the methodological quality of prognostic cohort studies on shoulder disorders

Criteria		Score
*Study population*		
A.	Inception cohort (defined in relationship to onset of symptoms)	+/−/?
B.	Description of inclusion and exclusion	+/?
C.	Description of study population	+/?
*Response*		
D.	Response >75 %	+/−/?
E.	Information about non-responders versus responders	+/−/?
*Follow-up (extent and length)*		
F.	Prospective data collection	+/−/?
G.	Follow-up of at least 6 months	+/−/?
H.	Drop-outs/loss to follow-up < 20 %	+/−/?
I.	Information completers versus loss to follow-up/drop-outs	+/−/?
*Treatment*		
J.	Treatment in cohort is fully described/standardised	+/−/?
*Outcome*		
K.	Standardised assessment of relevant outcome criteria	+/?
*Prognostic factors*		
L.	Standardised assessment of patient characteristics and potential clinical prognostic factor(s)	+/?
M.	Standardised assessment of potential psychosocial prognostic factor(s)	+/?
*Data presentation*		
N.	Frequencies of most important outcome measures	+/−
O.	Frequencies of most important prognostic factors	+/−
P.	Appropriate analysis techniques	+/−/?
Q.	Prognostic model is presented	+/−/?
R.	Sufficient numbers	+/−

### Analysis

Data were extracted by using a predefined data extraction form regarding study population, design, setting, outcome measures, prognostic factors and strength of association. To facilitate interpretation and comparison of the results the studies were categorized per setting (primary care, secondary care and occupational setting). Statistically significant multivariate associations or if not available, univariate associations were presented. Non-significant associations were summarised. Prognostic factors examined only once were described separately from those occurring twice or more. Classification of prognostic factors was performed independently by two reviewers (MK and DB), if necessary, a third (IS) and fourth (CV) reviewer were consulted until consensus was reached. Outcome measures where so diverse that we chose to organize them in either ‘better’ or ‘poorer’ outcome. For example, less pain, better function, being able to work and no recurrent complaints were considered ‘better’ and more pain, more disability and worse (perception of) outcome as ‘poorer’. Due to heterogeneity in study population, setting, prognostic factors and outcome measures, statistical pooling of results (meta-analysis) was considered inappropriate. Instead, a best evidence synthesis was performed. In this qualitative analysis, conclusions are based on the number of studies evaluating this factor, consistency of results and methodological quality (Table 2). Results were considered consistent if > 75 % of the studies reported results in the same direction [9, 10].

**Table 2:** Levels of evidence for prognostic factors on shoulder disorders

Level of evidence	
Strong	Consistent findings (>75 %) in at least two high quality cohorts
Moderate	Consistent findings (>75 %) in one high quality cohort and at least one low quality cohort
Weak	Findings of one high quality cohort or consistent findings (>75 %) in at least three or more low quality cohorts
Inconclusive	Inconsistent findings irrespective of study quality, or less than three low quality cohorts available
No evidence	No data presented

## Results

### Selection of studies

**Fig. 1:**
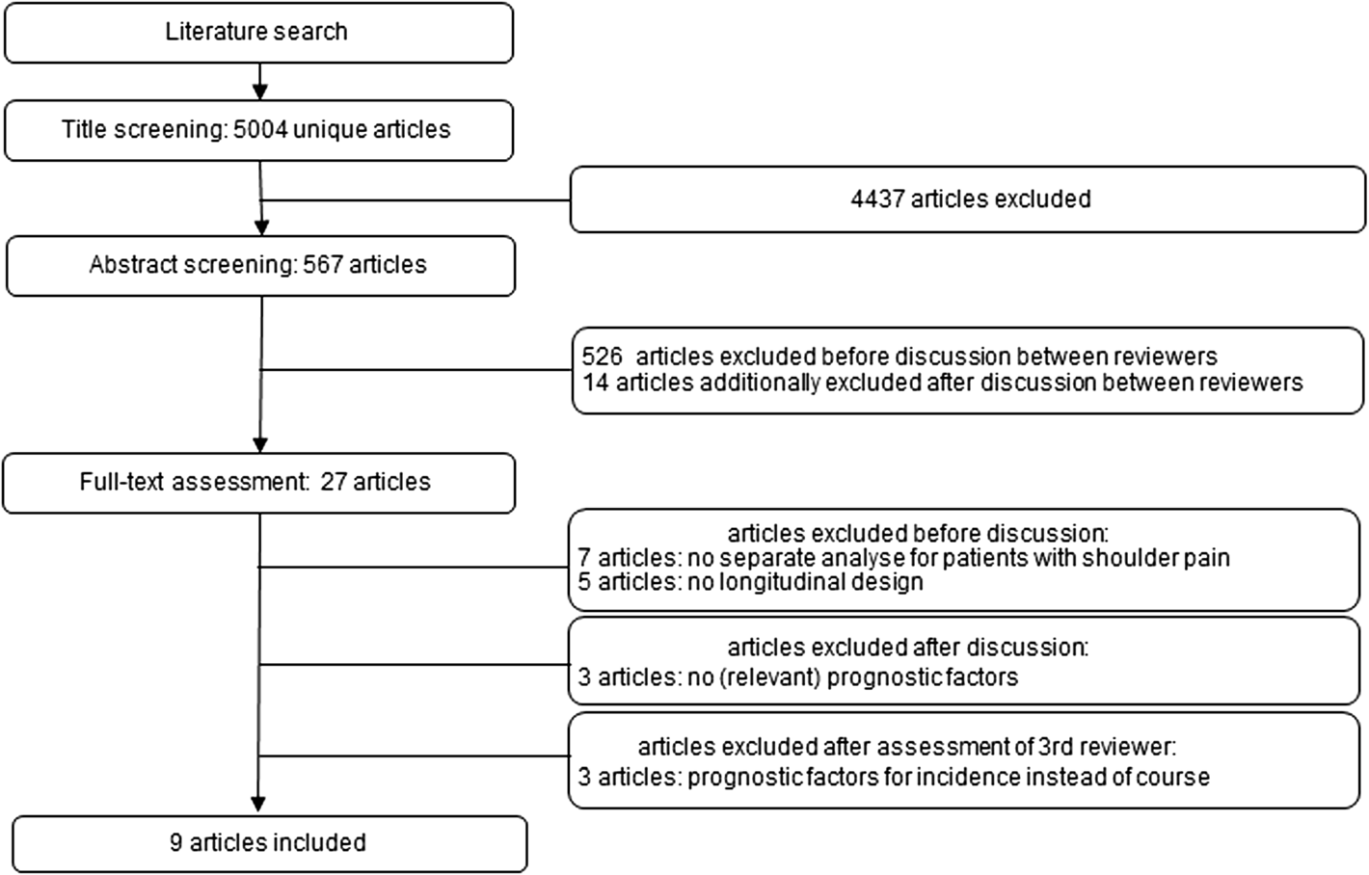
Overview of the selection procedure

The literature search yielded 5,004 citations. After completion of the selection procedure, 4,995 publications were eliminated based on title, abstract and full-text, leaving nine studies of which the methodological quality was assessed [11–19]. Figure 1 (flowchart) shows an overview of the study selection procedure.

### Methodological quality

There was disagreement between reviewers on seven of 162 (9 × 18) items (4 %), which was solved by discussion. Six studies were classified as high quality studies and three as low quality, there was a range in scores between 9 and 16 points. Table 3 presents the methodological quality of all studies, including those of the original review. In most studies, items ‘description of study population’ (C), ‘prospective data collection’ (F), ‘assessment of outcome criteria’ (K), ‘assessment of patient characteristic and prognostic factors’ (L), ‘frequencies of outcome measures’ (N) and ‘frequencies of prognostic factors’ (O) of the criteria list were well described. In five studies, follow-up was shorter than six months (G). Both items on response were poorly described; in eight studies the response rate was higher than 75 % (D) and in three studies information was given about responders/non responders (E). In addition, the minority of studies included information on drop-outs or those lost to follow up.

**Table 3:** Results of the methodological assessment of prognostic cohort studies on shoulder disorders

		A	B	C	D	E	F	G	H	I	J	K	L	M	N	O	P	Q	R	Quality score	Score (%)
First author																					
Bartolozzi	1994	-	+	-	?	?	+	+	-	?	+	+	+	?	+	+	+	-	+	10	56
Binder	1984	-	+	-	?	?	+	+	+	?	+	+	+	?	+	+	-	-	-	9	50
Brox	1996	-	+	+	+	-	?	+	+	+	?	+	+	+	+	+	+	+	+	14	78
Cassou	2002	+	+	+	+	?	+	+	+	?	?	+	+	+	+	+	+	+	+	15	83
Chard	1988	+	?	+	?	?	+	+	+	?	?	+	+	?	+	+	+	?	+	11	61
Croft	1996	+	?	+	?	?	+	+	-	+	?	+	?	?	+	-	+	?	+	9	50
Engebretsen	2010	-	+	+	+	?	+	+	+	+	+	+	+	+	+	+	+	+	-	15	83
Gill	2013	-	?	+	?	?	+	+	?	?	?	+	+	-	+	+	-	+	+	9	50
Herin	2012	-	?	+	?	?	+	+	?	?	?	+	+	+	+	+	-	+	+	10	56
Kaergaard	2000	?	?	+	+	?	+	+	-	-	?	+	?	+	-	-	?	?	-	6	33
Kennedy	2006	+	+	+	+	+	+	-	+	?	+	+	+	+	+	+	+	+	+	16	89
Kuijpers	2006	+	+	+	?	?	+	+	+	+	+	+	+	+	+	+	+	+	+	16	89
Kuroda	2001	?	?	+	?	?	+	+	?	?	?	?	?	?	+	-	?	?	+	5	28
Luime	2004	-	?	+	?	?	+	+	-	-	?	+	+	+	+	+	+	+	-	10	56
Macfarlane	1998	-	?	+	+	?	+	+	-	+	?	+	+	+	+	+	+	+	+	13	72
Miranda	2001	?	?	+	+	+	+	+	?	?	?	+	+	+	+	-	+	+	+	12	67
Morrison	1997	?	+	-	?	?	+	+	+	?	+	+	+	?	+	+	-	-	+	10	56
Mulcahy	1994	-	?	+	?	?	+	-	-	?	+	+	?	?	?	-	-	-	-	4	22
O’Malley	2004	?	?	+	?	?	+	-	-	+	+	+	+	+	+	+	+	+	-	11	61
Shaffer	1992	-	+	+	?	?	+	+	-	?	-	+	?	?	+	-	-	-	-	6	33
Solomon	2001	-	+	+	?	?	+	+	?	?	?	+	+	?	-	+	+	+	-	9	50
Thomas	2004	+	+	+	?	?	+	+	+	?	+	+	+	?	+	+	+	+	+	14	78
Viikari	2000	+	?	+	-	+	+	-	?	?	?	+	?	?	+	+	+	+	+	10	56
Windt	1996	+	+	+	+	-	+	+	+	-	-	+	+	?	+	+	+	+	+	14	78
Windt	2007	+	+	+	?	?	+	-	+	+	+	+	+	+	+	+	+	+	+	15	83

### Characteristics of studies

Study characteristics are presented in Additional file 2. Eight studies were conducted in a primary care setting; ten in a secondary care setting and seven in an occupational setting. In total, 60 potential prognostic factors were evaluated. Pain, duration of symptoms, disability, age, gender and psychological factors were reported on most often. In all new studies, through multivariable analysis, an attempt was made to determine a set of prognostic factors with the highest prognostic value. Many studies conducted their analyses on more than one or on a combined outcome measure. This resulted in a wide variety of outcome measures including pain, disability, range of movement, patient perceived recovery, shoulder instability, recovery and several shoulder questionnaires combining these measures.

### Evidence for prognostic factors

A best evidence synthesis was performed to summarize prognostic factors of shoulder disorders. This included the results of the previous systematic review on this topic by Kuijpers et al. (2004) [8].

In Table 4, prognostic factors studied at least twice and their relationship with outcome are presented. It shows that there is strong evidence that higher shoulder pain intensity, concomitant neck pain and a longer duration of symptoms predict poorer outcome in primary care settings. In secondary care populations, strong evidence was found for the association between greater disability and poorer outcome and between the existence of previous shoulder pain and poorer outcome. In this population there is moderate evidence that higher education is associated with better outcome.

**Table 4:** Overall level of evidence for prognostic factors and their association with outcome

Prognostic factor assessed at baseline	Outcome	QS > 60 %	QS ≤ 60 %	Level of evidence
*Primary care*				
Higher shoulder pain intensity [5, 14, 15, 18, 22]	Poorer	4/5	─	Strong
	Better	1/5	─	
Concomitant neck pain [5, 15, 18]	Poorer	3/3	─	Strong
Longer duration of symptoms [14, 15, 18, 22, 23]	Poorer	4/4	1/1	Strong
Precipitating cause (trauma) [5, 15]	Better	1/2	─	Inconclusive
	No association	1/2	─	
Greater disability [14, 15, 18, 22, 23]	Poorer	2/4	1/1	Inconclusive
	No association	2/4	─	
Previous episode of pain [14, 15, 23]	Poorer	─	1/1	Inconclusive
	No association	2/2	─	
Female gender [5, 14, 15, 18, 22]	Better	1/5	─	Inconclusive
	Poorer	1/5	─	
	No association	3/5	─	
Gradual onset [14, 15, 18, 22]	Poorer	2/4	─	Inconclusive
	No association	2/4	─	
*Secondary care*				
Greater disability [11, 17, 24, 25]	Poorer	2/2	1/2	Strong
	Better	─	1/2	
No previous shoulder pain [11, 17]	Better	2/2	─	Strong
Higher education [11, 25]	Better	1/1	1/1	Moderate
Gradual onset [24, 26, 27]	Poorer	─	1/3	Inconclusive
	No association	─	2/3	
Long duration of complaints [11, 24, 26–28]	Poorer	─	2/4	Inconclusive
	No association	1/1	2/4	
Non-dominant side involved [24, 26–28]	Better	─	1/4	Inconclusive
	No association	─	3/4	
Diagnosis (large tear) [17, 24, 25, 29]	Poorer	─	1/3	Inconclusive
	No association	1/1	2/3	
Physical workload (manual work) [11, 28]	Poorer	─	1/1	Inconclusive
	No association	1/1	─	
Health status [11, 17]	Better	1/2	─	Inconclusive
	No association	1/2	─	
*Occupational setting*				
Longer duration of symptoms [16, 30]	Poorer	─	2/2	Inconclusive
Higher age [12, 13, 16, 31, 32]	Poorer	2/2	1/3	Inconclusive
	No association		2/3	
Female gender [12, 13, 16, 32]	Better	─	1/3	Inconclusive
	Poorer	─	1/3	
	No association	1/1	1/3	
Work related psychosocial factors [16, 31, 32]	Poorer	2/2	─	Inconclusive
	No association	─	1/1	
High physical workload [13, 16, 30–34]	Poorer	1/2	1/5	Inconclusive
	No association	1/2	3/5	
Sporting activities [13, 16, 31, 32, 34]	Better	─	1/3	Inconclusive
	Poorer	1/2	─	
	No association	1/2	2/3	

Table 5 gives an overview of prognostic factors studied at least twice that have shown no association with outcome. It shows that there is strong evidence that range of motion, age, psychological factors, education, comorbidity, muscle strength, dominance and medication use do not predict outcome in primary care populations. Body Mass Index appears not to be associated with outcome in occupational populations and gender, age, previous physiotherapy, GP treatment, psychological factors and, to a lesser extent, range of motion show no relationship with outcome in secondary care populations.

**Table 5:** Overall level of evidence for prognostic factors with no association with outcome

Prognostic factor assessed at baseline	Outcome	QS > 60 %	QS ≤ 60 %	Level of evidence
*Primary care*				
Restricted range of motion [14, 15, 22, 23]	Poorer	─	1/1	Strong
	No association	3/3	─	
Younger age [5, 14, 15, 18, 22]	Better	1/5	─	Strong
	No association	4/5	─	
Comorbid psychological factors [5, 14, 15, 22, 35]	No association	5/5	─	Strong
Education [15, 35]	No association	2/2	─	Strong
Comorbidity [11, 27]	No association	2/2	─	Strong
Muscle strength [14, 35]	No association	2/2	─	Strong
Dominance [5, 15, 18]	No association	3/3	─	Strong
Medication use [14, 18]	No association	2/2	─	Strong
*Secondary care*				
Gender [11, 17, 24–26, 28, 36]	No association	2/2	5/5	Strong
Older age [11, 17, 24–28, 36]	Poorer	─	1/6	Strong
	No association	2/2	5/6	
Previous physiotherapy [11, 17]	No association	2/2	─	Strong
GP treatment (medication) [11, 17]	No association	2/2	─	Strong
Psychological factors [11, 17]	No association	2/2	─	Strong
ROM [11, 24]	No association	1/1	1/1	Moderate
*Occupational setting*				
BMI [12, 13, 16, 32]	No association	1/1	3/3	Strong

## Discussion

A few conclusions can be drawn from this update of the literature on prognostic factors on shoulder disorders. In primary care populations, higher shoulder pain intensity, concomitant neck pain and a longer duration of symptoms seem to show an association with a poorer outcome whilst range of motion, age, psychological factors, education, comorbidity, muscle strength, arm dominance and medication use do not seem to be associated with outcome. In occupational populations it is less evident which prognostic factors are associated with outcome. Greater disability and the existence of previous shoulder pain show an association with a poorer outcome in secondary care population. In general, it is remarkable that most factors of prognostic importance are clinical variables.

This systematic review summarises 25 studies of which nine were published since the original review in 2004. Twelve studies were of high quality of which six were published since the original review. Relatively many new studies were conducted in primary care settings. This increase in studies conducted in primary care reflects reality much better since most patients only receive care from a general practitioner or a physiotherapist. However, only one study was conducted in physiotherapy practices, which limits the possibility for studying possible predictors of outcome in this specific setting.

A few findings, viz. on disability, pain, duration of the complaint and psychological factors, need further exploration. In spite of four high quality studies, there are conflicting results on the effect of baseline disability on outcome in primary care. This might be due to the number of outcome measures involved, which vary from solely pain to merely disability and several questionnaires incorporating both. The prognostic importance of pain seems to be more straightforward; more pain at baseline predicts poorer outcome. However, looking at the results in more detail, Thomas et al. (2005) showed that more severe pain was associated with more pain at follow-up but not with disability or general perceived recovery [18]. Kennedy et al. (2006) found that more pain was associated with *more* improvement in a combined pain/disability outcome measurement but not with absolute pain/disability at the end of treatment [14]. In addition, present review indicates these associations are different in secondary care; in this setting, more severe disability is related to poorer outcome and the evidence on pain is inconclusive. As a result, conclusions on pain and disability as a prognostic indicator for outcome seem prone to several factors and need to be interpreted with some caution. For duration of the complaints, in secondary care the evidence is conflicting but consists of four low and just one high quality study in which duration is not associated with outcome. The latter is easily explained because only patients with chronic shoulder complaints were included so little variation could be expected. Also in primary care quite some people wait long before they seek help for shoulder pain and this distribution is reflected in research. However, included studies do contain patients with acute, sub-acute and chronic complaints and reveal that there is very strong evidence that longer duration is associated with poorer outcome. Many clinicians may endorse this finding from clinical experience. As for psychological factors, in recent years this has been the subject or special interest of many studies. Although it is a broad construct including an array of psychological traits, present summary of the literature suggests that they have no clear association with outcome in either primary or secondary care settings.

A limitation of current study is that some predictors have become quite broad in definition, increasing the risk on finding conflicting evidence on their relationship with outcome. This grouping did make it possible to give an overview of factors that have no prognostic importance or have not been investigated often enough. Also outcome measures were very diverse and often consisted of a combination of several things at once, such as the SPADI, DASH and UCLA questionnaires, which measure pain and disability and some also range of motion, strength and/or patient satisfaction. Since pain and disability are the most common outcome measures, the choice was either to exclude studies in which other measures were used leaving the problem of combined measures, or to classify outcome as better or poorer. The authors agreed on this simplification, aware of the loss of nuance that might be relevant to the individual patient and clinician. Included tables should provide them with more detailed information or the reference as to where to find it.

For future research, we recommend to carry out more research in physiotherapy practices since only one study was conducted in this setting, which indicates that the influence of age, gender, onset and pain on outcome in this setting might be different from general practice. Even more so since these complaints are very common and in many countries patients do not need a referral from a physician (anymore) to visit a physiotherapist. Kuijpers et al. (2004) uncovered the need for well-conducted prospective cohort studies [8]. Those published since are indeed of much higher quality and the prognostic factors, however many, much better described. However, regardless of the setting, before starting new studies, researchers should consider the wide variety in outcome measures that exists which hamper synthesis of results. In our opinion, research into patient reported outcome measures (PROM’s) is useful here since PROM’s not only reflect the patients’ perception but also because when standardized, they facilitate comparison between studies. The methods for conducting systematic reviews of studies regarding prognostic questions itself are still in development, as well as a system for rating the quality of a body of evidence. In the future the GRADE system, which is widely used for questions regarding interventions, will be available for the subject of prognosis as well [20, 21].

There are some implications for clinical practice as well. From previous research it is known that patients with shoulder problems are mainly treated in primary care by general practitioners or physiotherapists. Present review shows that pain severity, concomitant neck pain and duration of symptoms have prognostic value for outcome in primary care settings. Since these are clinical variables that can be influenced, clinicians may take these factors into account in the management of their patients. Whereas current Dutch guidelines for general practitioners advise a wait-and-see policy for all patients with shoulder pain at first, they may decide to monitor those patients with a worse prognosis more frequently and alter the treatment plan timely if complaints persist.

## Conclusions

Present review shows that there is strong evidence that higher shoulder pain intensity, concomitant neck pain and a longer duration of symptoms predict poorer outcome in primary care settings. In secondary care populations, strong evidence was found for the association between greater disability and poorer outcome and between the existence of previous shoulder pain and poorer outcome. Since these are clinical variables that can be influenced, clinicians may take these factors into account in the management of their patients.

## Additional files

Additional file 1:
**Explanation of the criteria from Table 1.** (DOCX 106 kb) Additional file 2: Table S1. Summary of study characteristics of prognostic cohort studies on shoulder disorders. (DOCX 73 kb)
