# Supplementary material for: Pain intensity, neck pain and longer duration of complaints predict poorer outcome in patients with shoulder pain – a systematic review
Source: BMC Musculoskelet Disord. 2015 Oct 9;16:288. doi: 10.1186/s12891-015-0738-4 (PMC4600288; doi:10.1186/s12891-015-0738-4)
Supplement: Additional file 1: — Explanation of the criteria from Table 1. (DOCX 106 kb) [file 12891_2015_738_MOESM1_ESM.docx]

Appendix A. Explanation of the criteria from Table 1

A. Positive if patients were identified at an early uniform point (inception cohort) in the course of their disease (first episode, with restriction to duration of symptoms, of shoulder pain in lifetime or first treated episode of shoulder pain).

B. Positive if criteria were formulated for at least: age, duration of symptoms, relevant co-morbity (i.e cervical radiculopathy, luxation)/systemic diseases.

C. Positive if was described in what setting the patients were recruited (i.e. general practice, hospital, occupational setting).

D. Positive if the response rate was **≥** 75%.

E. Positive if information was presented about patient/disease characteristics of responders and non-responders or if there was no selective response.

F. Positive if a prospective design was used, also positive in case of an historical cohort in which the

determinants had been measured before outcome was determined.

G. Positive if the follow-up period was at least 6 months.

H. Positive if the total number of participants was **≥** 80% on the last moment of follow-up compared to the number of participants at baseline.

I. Positive if demographic/clinical information (patient/disease characteristics such as age, sex and other potential prognostic predictors) was presented for completers and those lost to followup/ drop-outs at the main moment of outcome measurement, or no selective drop-outs/lost to follow up, or no drop-outs/lost to follow-up.

J. Positive if treatment subsequent to inclusion in cohort is fully described or standardised. Also positive in case of no treatment given.

K. Positive if standardised questionnaires or objective outcome measurements of at least 1 of the following 5 outcome measures were used for each follow-up measurement: pain, general improvement, functional status, general health status or lost days of work.

L. Positive if standardised questionnaires or objective measurements were used at baseline for at least 4 of the following 8 potential prognostic factors: age, sex, pain, functional status, duration of complaints, neck complaints, physical workload, or dominant shoulder affected.

M. Positive if standardised questionnaires or objective measurements were used at baseline of at least 1 of the following 6 potential prognostic factors: depression, somatisation, distress, fear and avoidance, coping strategies, or psychosocial workrelated factors (social support, psychological demands, and job decision latitude).

N. Positive if frequency, percentage or mean, median (Inter Quartile Range) and standard deviation/CI (confidence interval) were reported for the most important outcome measures.

O. Positive if frequency, percentage or mean, median (Inter Quartile Range) and standard deviation/CI

were reported for the most important prognostic factors.

P. Positive if univariate crude estimates were provided for the association of a prognostic factor with outcome.

Q. Attempt is made to determine a set of prognostic factors with the highest prognostic value.

R. Positive if the number of cases in the multivariate analysis was at least ten times the number of independent variables in the analysis (Altman, 1991).
