# Supplementary material for: Pain intensity, neck pain and longer duration of complaints predict poorer outcome in patients with shoulder pain – a systematic review
Source: BMC Musculoskelet Disord. 2015 Oct 9;16:288. doi: 10.1186/s12891-015-0738-4 (PMC4600288; doi:10.1186/s12891-015-0738-4)
Supplement: Additional file 2: Table S1. — Summary of study characteristics of prognostic cohort studies on shoulder disorders. (DOCX 73 kb) [file 12891_2015_738_MOESM2_ESM.docx]

| Author | Study | Study population | Duration follow-up/ | Prognostic factor(s) | Strength of association (95% CI) |
| --- | --- | --- | --- | --- | --- |
|  | quality | | outcome measure(s) | |  |
|  | (%) |  |  |  |  |
|  |  |  |  |  |  |
| *Primary care* | |  |  |  |  |
|  |  |  |  |  |  |
| Brox and Brevik (1996) | 78 | 125 patients | 6 months |  | Multivariate analysis, adjusted for age, gender, symptom duration, baseline Neer score |
|  |  | Rotator tendinosis  Referred by general practitioner | Neer shoulder score (0–100)  Succes: ≥80 points | Not on sick leave | OR = 4.4 (1.6, 12.1) |
|  |  |  |  | Not on regular medication | OR = 4.2 (1.5, 11.1) |
|  |  |  |  | Active treatment (ref = not active) | OR = 4.8 (1.7, 13.6) |
|  |  |  |  | Years of education, overhead work activity, comorbidity, isometric strength endurance, locus of control beliefs, emotional distress | n.s. |
|  |  |  |  |  |  |
| Croft et al. (1996) | 50 | 166 patients | 6 months |  |  |
|  |  | Shoulder pain General practice | Validated 22-item disability questionnaire | Baseline disability score >10, symptom duration (>1 month), injection at baseline, previous episodes of shoulder pain, severely restricted passive elevation (<101°). | Poorer outcome (P < 0.05) (Beta’s not presented) |
|  |  |  |  |  |  |
| Kennedy et al. (2006) | 89 | 361 patients | max 12 weeks |  | Multivariate analysis (higher log 1 + DASH at discharge) |
|  |  | Shoulder complaints Physiotherapy practice | DASH at discharge (higher DASH is greater disability) | Age (older) | *β_by decade_*= 0.13 (0.06, 0.19), p = 0.0004 |
|  |  |  |  | Gender (female) | *β* = -0.24 (-0.45, -0.04), p = 0.0219 |
|  |  |  |  | Function/disability (baseline DASH) | *β* = 0.02 (0.01, 0.03), p < 0.0001 |
|  |  |  |  | Workers'compensation claim | *β* = 0.55 (0.18, 0.92), p = 0.0036 |
|  |  |  |  | Therapist predicts more restricton of patient return to usual activity | *β* = 0.42 (0.21, 0.63), p < 0.0001 |
|  |  |  |  |  |  |
|  |  |  |  | Onset, duration of pretreatment symptoms, familiair with shoulder complaints, pain (intensity), pain (global rating injury/pain), pretreatment ROM (restriction), pretreatment weakness (muscle strength), general health (SF 36 physical), additional medical problems (co-morbidity), work status, surgery, GP treatment (prescribed medication), psychological factors (SF 36 mental), patient outcome expectancy (patient prediction recovery) | n.s. |
|  |  |  |  |  |  |
|  |  |  | Change in DASH (larger improvement) | Age (younger) | *β_by decade_*= 1.58 (0.21, 2.96), p = 0.0243 |
|  |  |  |  | Duration of pretreatment symptoms (shorter) | *β* = 4.04 (1.60, 6.47), p = 0.001 |
|  |  |  |  | Pain (higher intensity, NRS 10-100)) | *β* = -1.56 (-2.56, -0.57), p = 0.02 |
|  |  |  |  | General health (worse SF 36) | *β* = 0.25 (0.01, 0.05), p = 0.0448 |
|  |  |  |  | Surgery (past six months) | *β* = -15.73 (-22.55, -8.9), p < 0.0001 |
|  |  |  |  |  |  |
|  |  |  |  | Gender, onset, familiair with shoulder complaints, pain (global rating injury/pain), function/disability (baseline DASH), pretreatment ROM (restriction, pretreatment weakness (muscle strength), additional medical problems (co-morbidity), work status, GP treatment (prescribed medication), psychological factors (SF 36 mental), patient outcome expectancy (patient prediction recovery) function/disability (baseline DASH) | n.s. |
|  |  |  |  |  |  |
| Kuijpers et al. (2006) | 89 | 587 patients | 6 months |  | Multivariate analysis |
|  |  | Shoulder pain General practice | Persistent symptoms at 6 weeks | Onset (gradual) | OR = 1.8 (1.1, 2.9) |
|  |  |  |  | Duration of pretreatment symptoms | OR_7-12/0-6weeks_ = 1.9 (1.1, 3.3) |
|  |  |  |  |  | OR_>12/0-6weeks_ = 2.6 (1.5, 4.4) |
|  |  |  |  | Pain (0-10) | OR = 1.1 (1.0, 1.2) |
|  |  |  |  | Neck problems (pain, 0-18) | OR = 1.1 (1.0, 2.7) |
|  |  |  |  | Repetitive movements | OR = 2.0 (1.2, 3.1) |
|  |  |  |  | Psychological factors (comorbid) | OR = 2.3 (0.9, 6.4) |
|  |  |  |  |  |  |
|  |  |  |  | Age, gender, education, dominance, precipitating cause, familiair with shoulder complaints, pain (with movement), function/disability, pretreatment ROM, additional musculoskeletal problems, physical activity, physical workload, psychological factors (coping, 4DKL, fear avoidance, kinesiophobia) | n.s. |
|  |  |  |  |  |  |
|  |  |  | Persistent symptoms at 6 months | Onset (gradual) | OR = 1.4 (1.0, 1.8) |
|  |  |  |  | Duration of pretreatment symptoms | OR_7-12/0-6weeks_ = 1.4 (0.9, 2.3) |
|  |  |  |  |  | OR_>12/0-6weeks_ = 1.9 (1.2, 3.0) |
|  |  |  |  | Pain (0-10) | OR = 1.1 (1.0, 1.2) |
|  |  |  |  | Additional musculoskeletal problems (low back pain) | OR = 1.6 (1.1, 2.5) |
|  |  |  |  | Shoulder pain physical examination | OR = 1.0 (1.0, 1.1) |
|  |  |  |  |  |  |
|  |  |  |  | Age, gender, education, dominance, precipitating cause, familiair with shoulder complaints, pain (with movement), function/disability, pretreatment ROM, neck problems (pain), neck problems (pain, ROM, with movement), physical activity, repetitive movements, physical workload, psychological factors (comorbid, coping, 4DKL, fear avoidance, kinesiophobia) | n.s. |
|  |  |  |  |  |  |
| Macfarlane et al. (1998) | 72 | 135 patients | 3 years |  | Multivariate analysis, adjusted for age and sex |
|  |  | Shoulder pain | % Shoulder pain | Pain at baseline | OR = 3.1 (1.1, 8.2) |
|  |  |  |  | Symptom duration (>1 year) | OR = 2.9 (1.1, 7.7) |
|  |  |  |  | Shoulder related disability (≥5 items on 22–item questionnaire) | OR = 3.1 (0.9, 11.0) |
|  |  |  |  |  |  |
|  |  |  |  | Age, sex, GP visit, area of pain, sudden onset, distress (GHQ), restricted ROM | n.s |
|  |  |  |  | , |  |
| Thomas et al. (2005) | 78 | 316 patients | 12-18 months |  | Multivariate analysis |
|  |  | Shoulder problems General practice | Severity of shoulder disability (higher) | Onset (gradual) | *β* = 7.77 (0.53, 15.0), p < 0.10 |
|  |  |  |  | Duration of pretreatment symptoms | *β* = 0.52 (0.08, 0.95), p < 0.10 |
|  |  |  |  | Function/disability (baseline, SDQ, per point) | *β* = 0.52 (0.36, 0.68), p < 0.10 |
|  |  |  |  | Neck problems (pain) | *β* = 6.57 (0.21, 12.90), p < 0.10 |
|  |  |  |  |  |  |
|  |  |  |  | Age, gender, dominance, pain, GP treatmet (use of painkillers in previous 48 hours) | n.s. |
|  |  |  |  |  |  |
|  |  |  | Pain severity (higher) | Gender (male) | *β* = 5.77 (0.74, 10.80), p < 0.10 |
|  |  |  |  | Duration of pretreatment symptoms | *β* = 0.30 (-0.06, 0.65), p < 0.10 |
|  |  |  |  | Pain (baseline, per point) | *β* = 0.13 (0.01, 0.28), p < 0.10 |
|  |  |  |  | Function/disability (baseline SDQ) | *β* = 0.15 (0.03, 0.26), p < 0.10 |
|  |  |  |  |  |  |
|  |  |  |  | Age, dominance, onset, neck problems, GP treatmet (use of painkillers in previous 48 hours) | n.s. |
|  |  |  |  |  |  |
|  |  |  | Patient perception of outcome (worsening) | Gender (male) | OR = 2.57 (1.10, 5.94), p < 0.10 |
|  |  |  |  | Onset (gradual) | OR = 3.21 (0.91, 11.3), p < 0.10 |
|  |  |  |  | Function/disability (baseline SDQ) | OR = 1.03 (1.01, 1.05), p < 0.10 |
|  |  |  |  |  |  |
|  |  |  |  | Age, dominance, duration of pretreatment smptoms, pain, neck problems, GP treatmet (use of painkillers in previous 48 hours) | n.s. |
|  |  |  |  |  |  |
| Van der Windt et al. (1996) | 78 | 349 patients | 12 months |  | Multivariate analysis |
|  |  | Shoulder pain  General practice | % Shoulder pain | Concomitant neck pain | OR = 2.8 (1.7, 4.6) |
|  |  |  |  | High pain intensity | OR = 2.0 (1.2, 3.3) |
|  |  |  |  | Precipitating trauma | OR = 0.4 (0.2, 0.9) |
|  |  |  |  | Diagnosis (acute bursitis) | OR = 0.4 (0.2, 0.8) |
|  |  |  |  |  |  |
|  |  |  |  | Age, gender, arm dominance | n.s. |
|  |  |  |  |  |  |
| Van der Windt et al. (2007) | 83 | 587 patients | 3 months |  | Multivariate analysis |
|  |  | Shoulder pain General practice | Perceived recovery (persistent symptoms) | Psychological factors (pain catastrophising, distress, somatisation, fear-avoidance) | n.s. |
|  |  |  |  |  |  |
|  |  |  | Functional disability (persistant disability) | Psychological factors (pain catastrophising, distress, somatisation, fear-avoidance) | n.s. |
|  |  |  |  |  |  |
| *Secondary care* |  |  |  |  |  |
|  |  |  |  |  |  |
| Bartolozzi et al. (1994) | 56 | 170 patients | Mean 20 months |  | Univariate analysis |
|  |  | Impingement syndrome  Orthopaedic dept | Shoulder rating system University of California Los Angeles (≥ 29 points) | Female | RR_female/male_ = 1.03 (0.81, 1.31) |
|  |  |  |  | Age | RR_40-60/<40y_ = 1.10 (0.78, 1.56) |
|  |  |  |  |  | RR_>60/<40y_ = 1.23 (0.89, 1.71) |
|  |  |  |  | Dominance | RR_non-dominant/dom_ = 0.95 (0.74, 1.24) |
|  |  |  |  | Onset of symptoms | RR_insidious/acute_ = 0.97 (0.75, 1.26) |
|  |  |  |  | Duration of pre-treatment symptoms* | RR_3-6 mnths/<3 mnths_ = 0.90 (0.72, 1.14) |
|  |  |  |  |  | RR_>6 mnths/<3 mnths_ = 0.69 (0.52, 0.91) |
|  |  |  |  | Functional impairment* | RR_moderate/mild_ = 0.81 (0.60, 1.09) |
|  |  |  |  |  | RR_severe/mild_ = 0.65 (0.46, 0.91) |
|  |  |  |  | Recreational or occupational demands | RR_moderate/low_ = 1.18 (0.85, 1.64) |
|  |  |  |  |  | RR_severe/low_ = 1.25 (0.97, 1.62) |
|  |  |  |  | Instability | RR_present/absent_ = 0.97 (0.64, 1.46) |
|  |  |  |  | ROM | RR_mild/none_ = 0.85 (0.60, 1.20) |
|  |  |  |  |  | RR_moderate/none_ = 0.78 (0.45, 1.36) |
|  |  |  |  |  | RR_severe/none_ = 0.96 (0.63, 1.46) |
|  |  |  |  | Weakness | RR_yes/no_ = 0.68 (0.49, 0.93) |
|  |  |  |  | Rotator cuff pathology:* Impingement or tendinitis | RR = 1.0 |
|  |  |  |  | Partial or small full thickness tear | RR = 0.82 (0.53, 1.26) |
|  |  |  |  | Moderate or large tear | RR = 0.34 (0.14, 0.80) |
|  |  |  |  | Treatment | No significant differences |
|  |  |  |  |  |  |
| Binder et al. (1984) | 50 | 42 patients | Mean 44 months |  | Mean difference abduction |
|  |  | Frozen shoulder  Patients Rheumatology dept | ROM | Non-dominant side involved  Manual work  Therapy: mobilisation versus injections, ice, or no additional treatment | Better: 12^0^ (P < 0.05)  Worse: 15^0^ (P < 0.05)  Worse: 15^0^ (P < 0.05) |
|  |  |  |  | Age, sex, symptom duration | n.s. |
|  |  |  |  |  |  |
| Engebretsen et al. (2010) | 83 | 104 patients | 12 months |  | Multivariate analysis adjusted for age and gender |
|  |  | Subacromial shoulder pain > 3 months Physical medicine and rehabilitation dept | SPADI (higher SPADI is greater disability) | Education level | *β_college, uni/<12 yrs school_*= -14.3 (-23.5, -5.2), p = 0.003 |
|  |  |  |  | Familiair with shoulder complaints | β = 11.0 (1.4, 20.6), p = 0.026 |
|  |  |  |  | Function/disability (baseline SPADI) | β= 0.37 (0.15, 0.59), p = 0.001 |
|  |  |  |  |  |  |
|  |  |  |  | Age, gender, duration of pretreatment symptoms, pain (intensity at rest), pretreatment ROM, neck problems (pain), general health (EQ-vas), work status, working above shoulder level, physical workload (frequency heavy lifting), previous physiotherapy, GP treatment (pain/sleeping/relax medication), psychological factors (emotional distess, self-efficacy for pain) | n.s. |
|  |  |  |  |  |  |
|  |  |  | Work status (working) | Function/disability (baseline SPADI) | OR*_college, uni/<12 yrs school_* = 4.3 (1.3, 14.9), p = 0.02 |
|  |  |  |  | General health (EQ-vas) | OR = 1.06 (1.0-1.1), p = 0.001 |
|  |  |  |  |  |  |
|  |  |  |  | Age, gender, education level, duration of pretreatment symptoms, familiair with shoulder complaints, pain (intensity at rest), function/disability (baseline SPADI), pretreatment ROM, neck problems (pain), work status, working above shoulder level, physical workload (frequency heavy lifting), previous physiotherapy, GP treatment (pain/sleeping/relax medication), psychological factors (emotional distess, self-efficacy for pain) | n.s. |
|  |  |  |  |  |  |
| Kuroda et al. (2001) | 28 | 341 patients | ≥ 3years |  | RR for recovery |
|  |  | Atraumatic shoulder instability  Shoulder disorder clinic (hospital) | Recovery | Stopping overhead sports | RR = 8.67 (2.7, 27.1) |
|  |  |  |  | Stopping non-overhead sports | RR = 1.37 (0.55, 3.43) |
|  |  |  |  | Female | RR = 0.94 (0.56, 1.58) |
|  |  |  |  | Age | P = 0.01 |
|  |  |  |  |  |  |
| O'Malley et al. (2004) | 61 | 199 patients | 3 months |  | Multivariate analysis |
|  |  | Shoulder problems Orthopedic dept | Shoulder function (better) | Familiar with shoulder complaints (no chronic conditions) | *β* = 3.23 (SE = 1.39), p = 0.02 |
|  |  |  |  | Function/disability (baseline) | *β* = 0.54 (SE = 0.12), p < 0.001 |
|  |  |  |  | Surgery (no surgery during study) | *β* = 6.76 (SE = 1.71), p < 0.001 |
|  |  |  |  | Patient outcome expectancy | *β* = 0.46 (SE = 0.14), p = 0.002 |
|  |  |  |  |  |  |
|  |  |  |  | Age, gender, pathology/diagnosis, general health (SF-12 physical), physical therapy (during study), previous physical therapy treatment, surgery (previous shoulder surgery), GP treatment (new medication during study), psychological factors (SF-12 mental) | n.s. |
|  |  |  |  |  |  |
|  |  |  | Patient outcome expectancy | Age | *β* = 0.03 (SE = 0.03), p = 0.29 |
|  |  |  |  | Function/disability (change) | *β* = 0.14 (SE = 0.06), p = 0.01 |
|  |  |  |  | Surgery (no previous surgery) | *β* = -0.24 (SE = 0.81), p = 0.77 |
|  |  |  |  | Patient outcome expectancy | *β* = 0.35 (SE = 0.10), p = 0.001 |
|  |  |  |  |  |  |
|  |  |  |  | Age, gender, familiar with shoulder complaints, pathology/diagnosis, function/disability (baseline), general health (SF-12 physical), physical therapy (during study), previous physical therapy treatment, surgery (previous shoulder surgery, surgery during study), GP treatment (new medication during study), psychological factors (SF-12 mental) | n.s. |
|  |  |  |  |  |  |
| Morrison et al. (1997) | 56 | 667 patients | Mean 27 months |  | Univariate analysis |
|  |  | Subacromial impingement syndrome  Center for sports medicine | Shoulder rating system University of California Los Angeles (≥28 points) | Female | RR = 0.97 (0.86, 1.09) |
|  |  |  |  | Dominance: dominant | RR = 1.0 |
|  |  |  |  | Non-dominant | RR = 0.92 (0.81, 1.05) |
|  |  |  |  | Bilateral | RR = 0.87 (0.67, 1.13) |
|  |  |  |  | Type Acromion: Type I | RR = 1.0 |
|  |  |  |  | Type II | RR = 0.74 (0.65, 0.84) |
|  |  |  |  | Type III | RR = 0.7 (0.61, 0.8) |
|  |  |  |  | Tenderness acromioclavicular joint Yes/no | RR = 0.83 (0.7, 0.98) |
|  |  |  |  | Age < 20 | RR = 1.0 |
|  |  |  |  | Age 21-40 | RR = 0.88 (0.7, 1.11) |
|  |  |  |  | Age 41-60 | RR = 1.0 (0.8, 1.25) |
|  |  |  |  | Age > 60 | RR = 0.76 (0.57, 1.01) |
|  |  |  |  | Onset: Acute | RR = 1.0 |
|  |  |  |  | Non-acute | RR = 0.81 (0.7, 0.94) |
|  |  |  |  | Chronic | RR = 0.86 (0.75, 0.99) |
|  |  |  |  |  |  |
| Mulcahy et al. (1994) | 22 | 51 patients | < 6 months |  |  |
|  |  | Hospital | Better, unchanged, worse | Tears (vs. no tears) | RR = 0.77 (0.47, 1.28) |
|  |  |  |  |  |  |
| Shaffer et al. (1992) | 33 | 92 patients | Mean 7 years |  |  |
|  |  | Frozen shoulder Orthopaedic clinic | ROM | Age, dominance, side,  acute or gradual onset,  minor trauma or spontaneous onset,  duration of symptoms at baseline, treatment,  response to treatment,  bilateral involvement,  associated medical problems | n.s. |
|  |  |  |  |  |  |
| Solomon et al. (2001) | 50 | 63 patients | 12 months | Multivariate associations: | Improvement pain / Improvement function |
|  |  | Acute shoulder pain  Hospital | Shoulder Pain and Disability Index | Referred | *β* = -2.4, p = 0.02 / *β* = -1.4, p = 0:17 |
|  |  |  |  | Worse baseline pain, per point | *β* = -4.2, p < 0.001 / Not in the model |
|  |  |  |  | Worse baseline function per point | Not in the model / *β* = -4.9, p < 0.001 |
|  |  |  |  | Older age per year | *β* = 1.2, p = 0.24 / *β* = -0.8, p = 0.46 |
|  |  |  |  | Female | *β* = 1.4, p = 0:2 / *β* = 1.3, p = 0.20 |
|  |  |  |  | More education, per year | *β* = 3.3, p = 0:0019 / *β* = 2.1, p = 0.047 |
|  |  |  |  | Longer pain duration per month | *β* = -2.2, p = 0:038 / *β* = -1.2, p = 0.24 |
|  |  |  |  | Osteoarthritis | *β* = 2.3, p = 0:026 / *β* = 1.0, p = 0.34 |
|  |  |  |  | Rotator cuff tear | *β* = -1.4, p = 0:026 / *β* = 1.1, p = 0.26 |
|  |  |  |  |  |  |
| *Occupational/general* | | |  |  |  |
|  |  |  |  |  |  |
| Cassou et al. (2002) | 83 | 1804 patients | 5 years | Year of birth (ref = 1953) | Multivariate analysis: Men / Women |
|  |  | Chronic neck-shoulder pain  Occupational physicians’ files | % Disappearance of pain | 1948 | OR = 1.5 (0.9, 2.5) / OR = 0.8 (0.5, 1.2) |
|  |  |  |  | 1945 | OR = 1.2 (0.8, 1.9) / OR = 0.6 (0.4, 0.8) |
|  |  |  |  | 1938 | OR = 1.0 (0.6, 1.5) / OR = 0.6 (0.4, 0.9) |
|  |  |  |  | Repetitive work (ref = never) |  |
|  |  |  |  | In 1990 | Univariate only / OR = 0.8 (0.5, 1.3) |
|  |  |  |  | Before 1990 | Univariate only / OR = 0.5 (0.3, 0.7) |
|  |  |  |  | High job demand | OR = 0.7 (0.5, 0.9) / OR = 0.7 (0.6, 0.9) |
|  |  |  |  | Previous musculoskeletal  disorders | OR = 0.4 (0.3, 0.6) / OR = 0.6 (0.5, 0.8) |
|  |  |  |  | Sporting activities | OR = 1.5 (1.1, 2.1) / Univariate only |
|  |  |  |  | Precise movements, awkward  work, repetitive work, job  control, shift work | n.s. / n.s. |
|  |  |  |  |  |  |
| Chard et al. (1988) | 61 | 137 | 19 months |  | Univariate analysis |
|  |  | Fotator cuff tendinitis  Shoulder clinic | Shoulder pain resolved | Precipitating cause: Unknown | RR = 1.0 |
|  |  |  |  | Injury | RR = 0.76 (0.40, 1.42) |
|  |  |  |  | Employment | RR = 0.63 (0.29, 1.40) |
|  |  |  |  | Precipitating Cause Overuse/strain | RR = 1.30 (0.82, 2.06) |
|  |  |  |  | Occupation  Housewife/retired | RR = 1.0 |
|  |  |  |  | Manual | RR = 0.88 (0.52, 1.51) |
|  |  |  |  | Non-manual | RR = 1.09 (0.67, 1.76) |
|  |  |  |  |  |  |
| Gill et al. (2013) | 50 | 408 patients | Median 4 years |  | Multivariate analysis adjusted f or age, sex & BMI |
|  |  | Shoulder complaints General population | Recurrent shoulder pain | Smoking | OR = 2.10 (1.19, 3.73), p = 0.011 |
|  |  |  |  | Depressive symptoms | OR 1.96 (1.07, 3.58), p = 0.029 |
|  |  |  |  | Knee pain | OR = 3.30 (2.09, 5.20), p < 0.001 |
|  |  |  |  | Hip pain | OR = 1.89 (1.10, 3.27), p = 0.022 |
|  |  |  |  | Back pain | OR = 3.88 (2.36, 6.37), p < 0.001 |
|  |  |  |  | Hand pain | OR = 2.77 (1.79, 4.29), p < 0.001 |
|  |  |  |  | Higher range of shoulder flexion | OR = 0.98 (0.97, 1.00), p = 0.027 |
|  |  |  |  | Higher range of shoulder abduction | OR = 0.98 (0.96, 0.99), p = 0.008 |
|  |  |  |  | Work status | OR_retired/employed_ = 0.47 (0.23, 0.99), p = 0.046 |
|  |  |  |  |  | OR_student/employed_ = 0.03 (0.01, 0.13), p < 0.001 |
|  |  |  |  |  |  |
|  |  |  |  | Age, gender, BMI, work status (part-time/casual, unemployed, home duties, other) | n.s. |
|  |  |  |  |  |  |
|  |  |  | Resolved shoulder pain | Gender (female) | OR = 3.21 (1.87, 5.52), p < 0.001 |
|  |  |  |  | Higher grip strength | OR = 1.04 (1.01, 1.07), p = 0.004 |
|  |  |  |  | Knee pain | OR = 1.68 (1.05, 2.69), p = 0.031 |
|  |  |  |  | Back pain | OR = 2.75 (1.79, 4.24), p < 0.001 |
|  |  |  |  | Hand pain | OR = 2.00 (1.19, 3.36), p = 0.009 |
|  |  |  |  | Higher range of shoulder abduction | OR = 0.97 (0.96, 0.98), p < 0.001 |
|  |  |  |  | Higher range of external rotation | OR = 0.98 (0.97, 1.00), p = 0.026 |
|  |  |  |  |  |  |
|  |  |  |  | Age, BMI | n.s. |
|  |  |  |  |  |  |
| Herin et al. (2012) | 56 | 734 patients | 5 years |  | Multivariate analysis adjusted for age, gender, sport, BMI and social class |
|  |  | Persistent chronic shoulder pain Under supervision of occupational physician | Persistent shoulder complaints | Age | OR_52/37years_ = 1.41 (1.03, 1.92), p = 0.03 |
|  |  |  |  | Gender (women/men) | OR = 1.50 (1.17, 1.94), p < 0.001 |
|  |  |  |  | Sporting activities | OR = 0.72 (0.58, 0.90), p < 0.001 |
|  |  |  |  | Posture | OR = 1.26 (1.00, 1.60), p = 0.05 |
|  |  |  |  |  |  |
|  |  |  |  | Body mass index, social class, phychological factors (psychological demand, decision latitude), biomechanical factors (forceful effort, effort with tools, heavy loads, movements, vibration) | n.s. |
|  |  |  |  |  |  |
| Kaergaard and Andersen (2000) | 33 | 40 | 2 years |  |  |
|  |  | Neck-shoulder disorders  Working population | % Recovery | Work exposure | n.s. |
|  |  |  |  | Physical activity at leisure time | n.s. |
|  |  |  |  |  |  |
| Luime et al. (2004) | 56 | 199 patients | max 2 years |  | Multivariate analysis |
|  |  | Shoulder complaints Workers of nursing homes | Recurrent complaints | Duration of pre-treatment symptoms (>3 months) | OR = 1.91 (1.36, 2.67) |
|  |  |  |  |  |  |
|  |  |  |  | Age, gender, BMI, general health, working above shoulder level, repetitive movements, physical activity, physical workload, psychological factors (low job control, high work demands, low supervisory support, low support from colleagues) | n.s. |
|  |  |  |  |  |  |
| Miranda et al. (2001) | 67 | 419 patients | 12 months | Individual factors: | Multivariate analysis |
|  |  | Shoulder pain Working population | % Persistent severe pain | Age < 35 | OR = 1.0 |
|  |  |  |  | Age 35 – 44 | OR = 0.9 (0.3, 2.6) |
|  |  |  |  | Age 45–54 | OR = 3.6 (1.3, 10.2) |
|  |  |  |  | Age ≥ 55 | OR = 1.6 (0.5, 4.8) |
|  |  |  |  | Sports activity added score > 156 vs < 52 | OR = 0.7 (0.4, 1.3) |
|  |  |  |  | Gender (female) | OR = 0.7 (0.4, 1.2) |
|  |  |  |  | Overload at work (definite vs none) | OR = 3.8 (1.8, 8.0) |
|  |  |  |  | Other work load factors (e.g.working above shoulder level), mental stress, body mass index | n.s. |
|  |  |  |  |  |  |
| Viikari-Juntura et al. (2000) | 56 | 474 patients | 60 days |  | Multivariate analysis |
|  |  | Neck-shoulder pain Occupational health service | Sick leave >3 days | Worker group (blue collar) | OR = 6.8 (2.1, 22.4) |
|  |  |  |  | Sick leave precedingexamination | OR = 6.5 (2.1, 20.4) |
|  |  |  |  | Symptom duration >7 days vs.0-2 days | OR = 0.1 (0.0, 0.3) |
|  |  |  |  | Continuous pain | OR = 1.7 (0.5, 5.7) |
|  |  |  |  | High pain intensity | OR = 1.1 (0.3, 4.0) |
|  |  |  |  | Interaction continuous pain x intensity | OR = 5.2 (1.0, 28.1) |
|  |  |  |  | Pain during rotation of the head | OR = 7.8 (3.0, 20.1) |
|  |  |  |  | Pain in shoulder in abduction of arm | OR = 5.9 (2.7, 12.7) |
|  |  |  |  | Other symptoms and signs | n.s. |
|  |  |  |  |  |  |
